# Hepatocellular Carcinoma With Different Areas of Right Retroperitoneal Space Invasion: Evaluation of Transcatheter Arterial Chemoembolization Efficacy and Blood Supply Characteristics

**DOI:** 10.3389/fonc.2020.539692

**Published:** 2020-09-23

**Authors:** Xi Liu, Guangsheng Liao, Xiaoping Luo, Wenlong Song, Haiping Zhang, Hao Chen, Shangzhi Cai, Dajing Guo

**Affiliations:** ^1^Department of Radiology, The Second Affiliated Hospital of Chongqing Medical University, Chongqing, China; ^2^Department of Radiology, Chongqing Traditional Chinese Medicine Hospital, Chongqing, China

**Keywords:** hepatocellular carcinoma, transcatheter arterial chemoembolization, extrahepatic collateral arteries, right retroperitoneal space, blood supply

## Abstract

**Purpose:**

To evaluate the therapeutic efficacy of transcatheter arterial chemoembolization (TACE) for hepatocellular carcinoma (HCC) with different areas of right retroperitoneal space (rRPS) invasion and analyze the blood supply.

**Methods:**

This retrospective study enrolled 41 patients with HCC with different areas of rRPS invasion treated with TACE, including 22 HCCs with superior aspect of the right perirenal space (SARPS) invasion and 19 HCCs with right anterior pararenal space (RAPS) invasion. The overall response rate (ORR) and disease control rate (DCR) were analyzed. The prognostic factors for overall survival (OS) after TACE were determined. The blood supply characteristics of HCC with different areas of rRPS invasion were analyzed with arteriograms.

**Results:**

All patients underwent 2.8 ± 1.8 TACE sessions over 25.0 ± 21.9 months. The median OS was 29.0 months for patients with SARPS invasion and 12.0 months for patients with RAPS invasion (*P* = 0.004). Only the invaded area of the rRPS was an independent prognostic factor for OS [hazard ratio (HR), 2.833; 95% CI, 1.297–6.188; and *P* = 0.009). The ORR and DCR were significantly higher in the group with SARPS invasion than in the group with RAPS invasion (ORR: 63.6% vs 31.6%, *P* = 0.041; DCR: 77.3% vs 47.4%, *P* = 0.047). Initially, HCC with SARPS invasion were supplied by the hepatic artery (HA; *n* = 8) and both the HA and extrahepatic collateral vessels (EHCs; *n* = 14); HCC with RAPS invasion were supplied by the HA (*n* = 10) and both the HA and EHCs (*n* = 9); as the TACE sessions increased, the tumor-feeding vessels shifted from the HA to both the HA and EHCs, and even EHCs could be the only blood supply. Rare EHCs appeared earlier and more frequently in the RAPS group than in the SARPS group.

**Conclusion:**

The efficacy of TACE differed for HCC with different areas of rRPS invasion, and the median OS, ORR and DCR were significantly better in the SARPS group than in the RAPS group. Different common EHCs supplied HCCs with different areas of rRPS invasion, while other rare EHCs appeared more frequently in the RAPS group.

## Introduction

Hepatocellular carcinoma (HCC) is the fifth most common malignancy worldwide and the third-most common cause of cancer mortality globally (1). More than half of HCC patients with extrahepatic invasion or macroscopic vascular invasion (MVI) are at an intermediate stage or advanced stage when diagnosed, so their treatment options may be limited, and the prognosis is very poor (2, 3).

The American Cancer Society reported that the 5-year survival rate of HCC that has spread to surrounding tissues or organs and/or regional lymph nodes is 11% (4). Once HCC invades the right retroperitoneal space (rRPS), patients often lose the chance to undergo surgery, and other treatment methods, such as ablation and high-intensity focused ultrasound (HIFU), are not effective. For unresectable HCC, especially intermediate-stage HCC, transcatheter arterial chemoembolization (TACE) is considered a standard and important treatment method (5, 6). However, the rates of post-treatment residual HCC remain high, and repeated TACE therapy is often needed (7, 8). One important factor that impacts the effect of TACE is insufficient suppression of the formation of extrahepatic collateral vessels (EHCs) in HCC (9, 10). These potential EHCs can seriously inhibit the effectiveness of TACE and lead to multiple recurrences.

In general, the anterior and upper areas of rRPS are invaded by HCC located in the posterior right lobe of the liver. The rRPS is a complicated three-dimensional space that is divided into three parts according to the right renal fascia, as follows: the right anterior pararenal space (RAPS), right perirenal space (RPS), and right posterior pararenal space (RPPS) (11, 12). Some previous studies have shown that the bare area of the liver and the RPS are connected by the superior aspect of the right perirenal space (SARPS) and there is no direct communication between the RAPS and the bare area of the liver (13–15). Because of the anatomical characteristics of the surrounding structures, HCC located in segment 7 of the liver mainly invades the RPS extrahepatically through the SARPS, and HCC located in segment 6 of the liver directly invades the RAPS extrahepatically.

Although many EHCs can supply the tumor tissue that invades the rRPS, such as the right inferior phrenic artery (RIPA), right adrenal artery (RAA), and right renal capsular artery (RRCA) (16), there is no systematic research on the efficacy of TACE and blood supply characteristics of HCC with different areas of rRPS invasion. Nonetheless, it is important to study the factors that influence treatment outcomes and to recognize the arterial blood supply of HCC with different areas of rRPS invasion to perform effective TACE.

The purpose of this study was to evaluate treatment response, identify independent predictive factors associated with overall survival (OS) after TACE, and further recognize the blood supply characteristics of HCC with SARPS and RAPS invasion.

## Materials and Methods

This study was approved by the ethics committee of our hospital, and the requirement for informed consent was waived. Informed consent for the TACE procedure was obtained from all patients before TACE treatment.

### Patients

Between May 2011 and August 2019, a total of 3567 TACE procedures were performed in 1635 HCC patients in our institution. We retrospectively analyzed newly developed HCC with different areas of rRPS invasion treated with superselective TACE. In the present study, the inclusion criteria, which were based on preoperative CT or MRI, were as follows: (1) protrusion of the inferior surface of segment 6 or segment 7 from the liver capsule; (2) no clear boundaries with adjacent organs, such as the adrenal gland, diaphragm, renal capsule and kidney, or pressure registered on adjacent organs; (3) blurred fat between the rRPS and HCC; and (4) first diagnosed without any previous HCC treatment. The exclusion criteria were as follows: (1) diffuse HCCs or extrahepatic metastasis that occurred before treatment; (2) Barcelona Clinic Liver Cancer (BCLC) terminal-stage HCC; (3) follow-up time of less than 1 month or irregular follow-up assessments; and (4) poor image quality. HCC was diagnosed based on typical imaging features as defined by the American Association for the Study of Liver Diseases (AASLD) guidelines (17). Consequently, a total of 41 patients (39 male and 2 female) with a mean age of 49.6 ± 10.3 years (range 31–70 years) were enrolled in our study, of whom 22 had HCC with SARPS invasion and 19 had HCC with RAPS invasion.

### Chemoembolization Procedure

For all patients, TACE was carried out with the same digital subtraction angiography machine (Infinix-i INFX-8000V, TOSHIBA, Japan) by two experienced interventional radiologists. TACE was performed via the transfemoral route with the Seldinger technique. The arteriograms of the celiac axis (CA) and superior mesenteric artery (SMA) were obtained at the beginning of the procedure with 5-F Rösch hepatic, 4-F Cobra or Yashiro catheters (Terumo Corporation, Tokyo, Japan). Angiographic images, including arterial, capillary, and venous phase images, were acquired to assess the tumor number, tumor location and possible types of EHCs, as well as the presence of vascular anatomic variations.

Based on the CT, MRI and angiographic images, the following observations were noted: (1) no tumor stain on angiography relative to that on CT or MRI; (2) hypertrophied RIPA, RRCA, and RAA or other EHCs that ran toward the tumor area on contrast-enhanced CT or MRI; and (3) defective iodized oil retention or progression of the peripheral area of the tumor after TACE therapy. We routinely performed examinations of the lateral or anterior wall of the abdominal aorta, right renal artery and subclavian artery and acquired arteriograms with 5 F Rösch hepatic, 4 F Cobra or Yashiro and 5 F Simmons catheters to look for EHCs. When the EHCs appeared to pass through the tumor stain on angiography, the tumor-feeding branch was superselectively cannulated by the microcatheter (Progreat Microcatheter System, 2.7 F, Terumo Corporation, Tokyo, Japan). Thus, treatment was implemented only after superselective catheterization of the tumor-feeding artery branches using a microcatheter, while avoiding non-target branches.

The TACE procedure was performed by infusing a mixture of iodized oil (Lipiodol; Andre Gurbet, Aulnay-sous-Bois, France) and anticancer drugs (10–50 mg cisplatin and 10–40 mg pirarubicin hydrochloride) before injecting the embolic material. The embolic materials were polyvinyl alcohol (PVA, size 150–350 μm, Alicon, Hangzhou, China) or gelatin sponge particles measuring 1 or 2 mm in diameter soaked in a mixture of non-ionic contrast medium.

The TACE procedures were the same as those initially performed as follow-up treatments.

### Therapeutic Efficacy Evaluation

Eighteen patients were evaluated with abdominal plain scan and enhanced CT, and 23 patients were evaluated with conventional and enhanced MRI within 1–3 months after initial TACE in our study. Two radiologists (XL and DG with 14 and 27 years of experience in abdominal CT and MRI) independently evaluated the efficacy retrospectively according to the modified Response Evaluation Criteria in Solid Tumors (mRECIST) criteria (18); if a consensus was not reached by both radiologists regarding the images, the images were evaluated by another senior radiologist. The treatment response, overall response rate (ORR), and disease control rate (DCR) were evaluated according to the mRECIST criteria. ORR was defined as the percentage of patients who achieved a complete response (CR) and a partial response (PR) among all eligible patients, and DCR was defined as the percentage of patients who achieved a CR, a PR, and a stable disease (SD) among all eligible patients. To assess OS, the patients were followed-up until the time of death or the end date of this study (August 31, 2019).

### EHC Assessment

The presence of an EHC supplying the tumor was defined as follows: (1) an obvious tumor stain was demonstrated on the arteriogram of the EHC during TACE; (2) a hypertrophied EHC running toward the tumor was observed; and (3) an arteriogram of the EHC during TACE showed a stain corresponding to the tumor area where defective iodized oil retention or progression of the peripheral area of the tumor was detected.

### Statistical Analysis

Overall response rate and DCR were compared between the two groups using the Pearson chi-square test. Univariate analysis was performed to identify the predictive factors. We used the Cox proportional hazards regression model and identified statistically significant variables in the univariate analysis (*P* < 0.1). Then, stepwise selection was used to identify the independent predictive factors by multivariate analysis. The OS after treatment was calculated by the Kaplan–Meier method and was compared using the log-rank test. All statistical analyses were performed using SPSS software version 26 (IBM Corporation, Armonk, NY, United States). A two-tailed *P* value < 0.05 was considered statistically significant.

## Results

### Patient Characteristics

The baseline characteristics of the patients are summarized in Table 1. A total of 41 patients underwent 2.8 ± 1.8 TACE sessions over 25.0 ± 21.9 months (range, 3–96 months). The median maximum tumor size was 8.4 ± 3.3 cm (range, 2.3–20.2 cm). 23 patients (56.1%) had liver cirrhosis, which was associated with hepatitis B in 22 patients (53.7%) and hepatitis C in 1 patient (2.4%). 30 patients (73.2%) were Child-Pugh class A, and 11 patients (26.8%) were Child-Pugh class B. Only 1 patient (2.4%) with BCLC stage A, 19 patients (46.3%) with BCLC stage B, and 21 patients (51.2%) with BCLC stage C HCC were enrolled in this study. 21 patients had portal vein (PV)/hepatic vein (HV) branch invasion, including 18 patients with PV branch invasion, 2 patients with right HV invasion, and 1 patient with PV branch and right HV invasion simultaneously, and all patients without main PV invasion. 22 patients (53.7%) had HCC with SARPS invasion via the inferior surface of segment 7, and 19 patients (46.3%) had HCC with RAPS invasion via the inferior surface of segment 6.

**TABLE 1:** Baseline clinical characteristics of the patients.

Characteristic	Value
Age (year)	49.6 ± 10.3 (31–70)
>50	17 (41.5%)
≤50	24 (58.5%)
Sex	
Male	39 (95.1%)
Female	2 (4.9%)
Child-Pugh classification	
A	30 (73.2%)
B	11 (26.8%)
BCLC stage	
A	1 (2.4%)
B	19 (46.3%)
C	21 (51.2%)
Liver cirrhosis	
Absent	18 (43.9%)
Present	23 (56.1%)
Etiology	
HBsAg	36 (87.8%)
HCV Ab	2 (4.9%)
Unknown	3 (7.3%)
No. of chemoembolizations	2.8 ± 1.8 (1–7)
Maximum tumor size (cm)	8.4 ± 3.3 (2.3–20.2)
>5 cm	35 (85.4%)
≤5 cm	6 (14.6%)
Multiplicity of tumor	
Single	31 (75.6%)
Multiple	10 (24.4%)
PV/HV branch invasion	
Absent	20 (48.8%)
Present	21 (51.2%)
Invaded area of the rRPS	
SARPS	22 (53.7%)
RAPS	19 (46.3%)
AFP (ng/mL)	
>400	26 (63.4%)
≤400	15 (36.6%)
Portal hypertension	
Absent	17 (41.5%)
Present	24 (58.5%)

*BCLC stage, Barcelona Clinic Liver Cancer stage; HBsAg, hepatitis B surface antigen status; HCV Ab, hepatitis C virus anti-body; PV, portal vein; HV, hepatic vein; rRPS, right retroperitoneal space; SARPS, superior aspect of the right perirenal space; RAPS, right anterior pararenal space; and AFP, alpha-fetoprotein.*

### Therapeutic Efficacy

**FIGURE 1:**
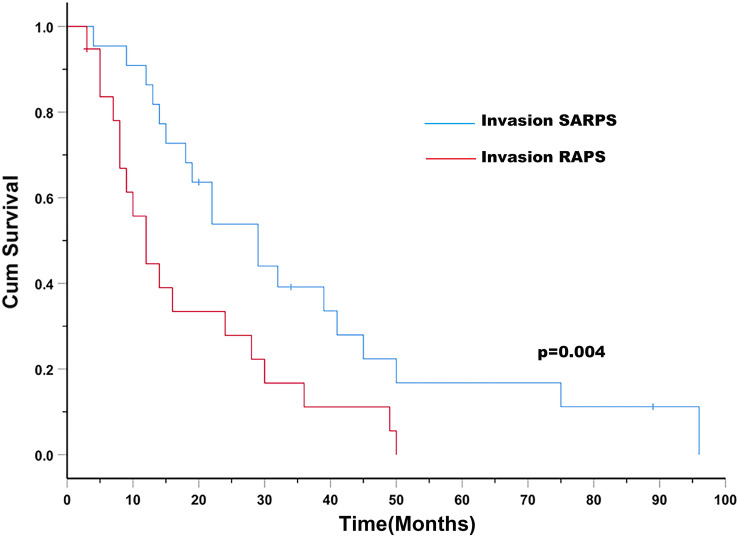
Patients with HCC with SARPS invasion (blue line) have an increased survival rate compared to those with HCC with RAPS invasion (red line; *P* = 0.004). Abbreviations: SARPS, superior aspect of the right perirenal space; RAPS, right anterior pararenal space.

The median OS was 19.0 months (95% CI: 11.7 months, 26.3 months) for patients with HCC with rRPS invasion. The median OS was 29.0 months (95% CI: 18.2 months, 39.8 months) for patients with HCC with SARPS invasion and 12.0 months (95% CI: 7.9 months, 16.1 months) for patients with HCC with RAPS invasion (*P* = 0.004; Figure 1). The invaded area of the rRPS, maximum tumor size, alpha-fetoprotein (AFP), presence of portal hypertension, and PV/HV branch invasion were significantly associated with OS in the univariate analysis (Table 2; *P* < 0.1). The invaded area of the rRPS was the only independent prognostic factor for OS using multivariate analysis (HR, 2.833; 95% CI, 1.297–6.188; and *P* = 0.009; Table 2).

**TABLE 2:** Univariate analysis and multivariate analysis of the clinical features that affect OS.

Factors	Univariate analysis		*P*	Multivariate analysis		*P*
	HR	OR (95% CI)		HR	OR (95% CI)	
Age	0.977	0.502–1.899	0.945			
Sex	2.476	0.561–10.926	0.231			
Invaded area of the rRPS	2.573	1.305–5.071	0.006	2.833	1.297–6.188	0.009*
Maximum tumor size	0.425	0.161–1.120	0.083	0.956	0.294–3.109	0.941
Number of tumors	1.093	0.448–2.666	0.845			
AFP (ng/mL)	0.353	0.169–0.740	0.006	0.444	0.178–1.108	0.082
Child-Pugh class	1.579	0.740–3.367	0.237			
BCLC stage	1.644	0.889–3.041	0.113			
Portal hypertension	1.841	0.916–3.702	0.087	1.154	0.537–2.481	0.713
Liver cirrhosis	1.430	0.708–2.887	0.318			
PV/HV branch invasion	1.753	0.907–3.385	0.095	1.670	0.786–3.547	0.182

*P values less than 0.05 were considered statistically significant. Significant variables with *P* < 0.1 in the univariate analysis were included in the multivariate Cox analysis, and the HR value is the Cox regression coefficient. Abbreviations: OS, overall survival; rRPS, right retroperitoneal space; AFP, alpha-fetoprotein; BCLC stage, Barcelona Clinic Liver Cancer stage; PV, portal vein; and HV, hepatic vein.

Among these 41 patients, the overall tumor responses were as follows: CR, *n* = 15 (36.6%); PR, *n* = 5 (12.2%); SD, *n* = 6 (14.6%); and progressive disease (PD), *n* = 15 (36.6%). The ORR and DCR were 48.9% and 63.4%, respectively. Of the 22 patients with HCC with SARPS invasion, the number of patients who achieved CR, PR, SD, and PD were 10 (45.5%), 4 (18.2%), 3 (13.6%), and 5 (22.7%), respectively. Among the 19 patients with HCC with RAPS invasion, the number of patients who achieved CR, PR, SD, and PD were 5 (26.3%), 1 (5.3%), 3 (15.8%), and 10 (52.6%), respectively. The ORR and DCR were significantly higher in the group with SARPS invasion than in the group with RAPS invasion (ORR: 63.6% vs 31.6%, *P* = 0.041; DCR: 77.3% vs 47.4%, *P* = 0.047).

### Characteristics of the Blood Supply

Since the invaded area of the rRPS was the only independent prognostic factor for OS, we analyzed the blood supply characteristics of each area, especially for the incidence of EHC supply. A summary of the blood supply to HCC with different areas of rRPS invasion is provided in Table 3.

**TABLE 3:** Summary of the blood vessels that supplied HCCs invading different areas of the rRPS.

	SARPS invasion	RAPS invasion	Total
HA	64	50	114 (45.8%)
RIPA	37	18	55 (22.1%)
RRCA	17	12	29 (11.6%)
RAA	24	4	28 (11.2%)
OA	2	5	7 (2.8%)
SA	1	4	5 (2.0%)
RPICA	2	2	4 (1.6%)
LA	0	2	2 (0.8%)
CA	1	1	2 (0.8%)
RA	0	1	1 (0.4%)
RAICA	1	0	1 (0.4%)
RCA	0	1	1 (0.4%)
Total	149	100	249

*HCC, hepatocellular carcinoma; rRPS, right retroperitoneal space; SARPS, superior aspect of the right perirenal space; RAPS, right anterior pararenal space; HA, hepatic artery; RIPA, right inferior phrenic artery; RRCA, right renal capsular artery; RAA, right adrenal artery; OA, omental artery; SA, supraduodenal artery; RPICA, right posterior intercostal artery; LA, lumbar artery; CA, cystic artery; RA, renal artery; RAICA, right anterior intercostal artery; and RCA, right colic artery.*

All tumors were partially or completely supplied by branches of the HA at the initial TACE procedures; the tumors in 18 patients (43.9%) were supplied by the HA alone, and the other tumors in 23 patients (56.1%) were supplied by both the HA and EHCs.

**FIGURE 2:**
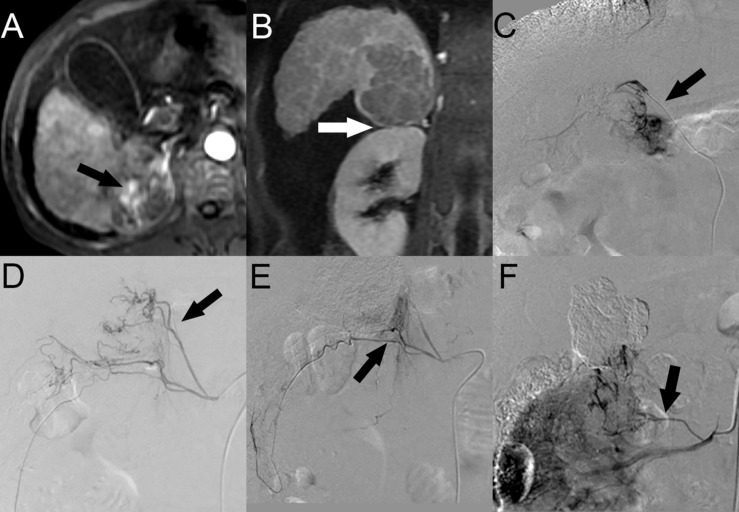
HCC with SARPS invasion in a 49-year-old man. **(A)** Contrast-enhanced arterial phase MR image showed a 5.4-cm-sized enhanced tumor (black arrow) in segment 7 of the right lobe of the liver in the axial plane. **(B)** Portal phase MR image showed clear tumor invasion of the SARPS in the coronal plane (white arrow). **(C)** Angiographic image at the first TACE session revealed that the tumor was supplied by the posterior branch of the RIPA (black arrow). **(D)** Angiographic image at the second recurrence revealed that the EHCs were supplied by the inferior branch of the RAA (black arrow). **(E)** After the inferior branch of the RAA was successfully chemoembolized, the angiogram showed that the superior branch of the RRCA was also involved in the tumor blood supply (black arrow). **(F)** Angiographic image at the fourth recurrence revealed that the tumor was supplied by the new inferior branch of the RAA (black arrow). Abbreviations: HCC, hepatocellular carcinoma; SARPS, superior aspect of the right perirenal space; RIPA, right inferior phrenic artery; RRCA, right renal capsular artery; RAA, right adrenal artery; and EHC, extrahepatic collateral vessel.

Twenty-two patients (53.7%) with HCC with SARPS invasion underwent a total of 3.3 ± 1.8 TACE sessions. A total of 149 blood vessels supplied the tumors; 8 tumors were supplied by the HA alone, and another 14 tumors were supplied by both the HA and EHCs at the initial TACE procedures. The major EHCs were the RIPA (*n* = 12), RAA (*n* = 7), and RRCA (*n* = 4). Of the 17 tumors that recurred once, 8 tumors were supplied by the HA alone, 6 tumors were supplied by both the HA and EHCs, and 3 tumors were supplied by EHCs alone. Of the 32 tumors that recurred two to six times during follow-up, 7 tumors were supplied by the HA alone, 21 tumors were supplied by both the HA and EHCs, and 4 tumors were supplied by EHCs alone. Angiography showed that the main EHCs were the RIPA (*n* = 25), RRCA (*n* = 13), and RAA (*n* = 17); as the number of TACE sessions increased, other EHCs were rarely observed, such as the omental artery (OA, *n* = 2) at the second and third recurrence, right posterior intercostal artery (RPICA, *n* = 2) at the third and sixth recurrence, supraduodenal artery (SA, *n* = 1) at the sixth recurrence, cystic artery (CA, *n* = 1) at the third recurrence, and right anterior intercostal artery (RAICA, *n* = 1) at the sixth recurrence (Figure 2).

**FIGURE 3:**
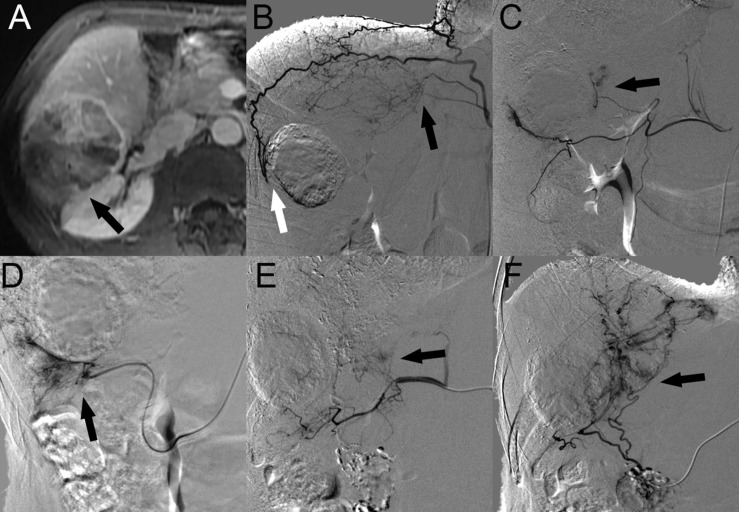
HCC with RAPS invasion in a 51-year-old man. **(A)** Contrast-enhanced portal phase MR image showed a 7.6-cm-sized tumor (black arrow) in segment 6 of the right lobe of the liver invading the RAPS in the axial plane. **(B)** Angiographic image at the first recurrence revealed that both the superior branch of the RAA (black arrow) and the posterior branch of the RIPA are involved in the tumor blood supply (white arrow). **(C)** Angiographic image at the TACE session to the first recurrence revealed that the tumor was also supplied by the middle branch of the RRCA (black arrow). **(D)** Angiographic image at the second recurrence revealed that the EHCs were supplied by the accessory omental artery (black arrow). **(E)** After the fourth chemoembolization session, the SA was involved in the tumor blood supply (black arrow). **(F)** At the last TACE session, the tumor was supplied by the right omental artery (black arrow). Abbreviations: HCC, hepatocellular carcinoma; RAPS, right anterior pararenal space; RIPA, right inferior phrenic artery; RRCA, right renal capsular artery; RAA, right adrenal artery; and SA, supraduodenal artery.

Nineteen patients (46.3%) with HCC with RAPS invasion underwent a total of 2.4 ± 1.7 TACE sessions. A total of 100 blood vessels supplied the tumor; 10 tumors were supplied by the HA alone, and another 9 tumors were supplied by both the HA and EHCs at the initial TACE procedures. The major EHCs were the RIPA (*n* = 6), RAA (*n* = 2), and RRCA (*n* = 4). In particular, 2 cases showed that the OA was involved in tumor blood supply. Of the 11 tumors that recurred once, 2 tumors were supplied by the HA alone, 9 tumors were supplied by both the HA and EHCs, and no tumors were supplied by EHCs alone. Of the 15 tumors that recurred two to six times, 4 tumors were supplied by the HA alone, 10 tumors were supplied by both the HA and EHCs, and 1 tumor was supplied by EHCs alone. During follow-up, angiography showed that the main EHCs were the RIPA (*n* = 12), RRCA (*n* = 8), RAA (*n* = 2), and OA (*n* = 3). As the number of TACE sessions increased, other EHCs were rarely observed, such as the SA (*n* = 4) at the third, fifth, and sixth recurrence, RPICA (*n* = 2) at the third and sixth recurrence, lumbar artery (LA, *n* = 2) at the third recurrence, CA (*n* = 1) at the third recurrence and renal artery RA (*n* = 1) at the second recurrence, and right colic artery (RCA, *n* = 1) at the fifth recurrence (Figure 3).

### Complications

In our study, most of the patients experienced common complications after TACE, including postembolization syndrome, liver function damage, and infection, but no serious complications such as intestinal ischemia and necrosis, ectopic embolization, or death of the patients occurred.

## Discussion

In our retrospective study, patients with HCC with rRPS invasion after TACE showed a median OS of 19.0 months without serious complications. This duration is shorter than that reported by Chung Young-Hwa et al. (19); however, considering that 21 (51.2%) patients in this study were classified as BCLC stage C and received only TACE treatment, this survival outcome is acceptable. We demonstrated that the median OS of the group with SARPS invasion was significantly longer than that of the group with RAPS invasion (29.0 months vs. 12.0 months; *P* = 0.004), and the ORR and DCR were significantly higher in the group with SARPS invasion than in the group with RAPS invasion (ORR: 63.6% vs 31.6%, *P* = 0.041; DCR: 77.3% vs 47.4%, *P* = 0.047).

Although TACE is a primary recommended treatment for HCC patients with stage B disease based on the BCLC staging system (5), many previous studies have confirmed that TACE treatment is safe and can benefit patients with BCLC stage C disease and main PV invasion if they have good liver function and a sufficient portal collateral circulation (20, 21). In our study, these patients had good liver function (Child-Pugh A/B:15/6) and no main PV invasion, so our institution performed TACE. A recent cohort study (22) of a multicenter registry database in Korea reported the prognosis of HCC patients with BCLC stage B or C disease and subdivided the patients by identifying prognostic predictors. The results showed that in the intermediate-stage HCC group, the median OS of the B1 (tumor size < 5 cm), B2 (tumor size ≥ 5 cm and Child-Pugh A), and B3 (tumor size ≥ 5 cm and Child-Pugh B) groups was 30.73, 20.60, and 9.23 months, respectively; in the advanced stage HCC group, the median OS of the C1 [neither significant portal vein invasion (sPVI) nor extrahepatic spread (EHS)], C2 (either sPVI or EHS), and C3 (both sPVI and EHS) groups was 8.43, 4.63, and 3.63 months, respectively. In our study, all patients had EHS, 35 (85.4%) patients had a tumor size > 5 cm, and 21 (51.2%) patients had vascular invasion. The survival outcomes of the B1 group were comparable to those of the group with SARPS invasion, which was in accordance with the report from Llovet et al. (23). The survival outcomes of the group with RAPS invasion were much better than those of the advanced stage HCC group. We consider that this might be related to the strict inclusion and exclusion criteria and adequate embolization of EHCs that supply HCC with rRPS invasion.

In terms of our related results, the invaded area of the rRPS, maximum tumor size, AFP, portal hypertension, and presence of PV/HV branch invasion were significant as prognostic factors for OS in the univariate analysis, which is consistent with literature reports (24–26). However, the Cox proportional hazards regression model indicated that only the invaded area of the rRPS was an independent prognostic factor for OS, which is consistent with the view that local recurrence is an independent prognostic factor for HCC in the literature (27). From another point of view, Shao et al. (28) reported that PV invasion was an independent prognostic factor for OS. This discrepancy might be mainly caused by the inclusion of all BCLC stage C HCC patients. Our study included a subset of patients (*n* = 19, 46.3%) with BCLC stage B HCC and nearly half of the patients (*n* = 20, 48.8%) did not have vascular invasion.

At the main endpoint of this study, we found that the median OS of the group with SARPS invasion was significantly longer than that of the group with RAPS invasion. In terms of secondary endpoints, overall, the ORR and DCR were only 48.9% and 63.4%, respectively, similar to the results described by Li et al. (29), which may be related to the large tumors (*n* = 35, >5 cm), PV/HV branch invasion, and retroperitoneal invasion in the patients we included. However, the ORR and DCR were significantly higher in the group with SARPS invasion than in the group with RAPS invasion, which we believe that this finding may be related to the location and aggressiveness of the tumors, since there were no notable differences in baseline characteristics among patients with HCC with different areas of rRPS invasion, we believe that these data were justified.

It is well known that the key to improving the efficacy of TACE is to accurately identify the tumor blood supply (30); in particular, the appearance of each collateral vessel in one specific session of TACE and the order of EHC occurrence throughout the TACE sessions is important (9, 10). Accordingly, our study is the first to systematically analyze the prognostic factors and blood supply characteristics of HCC with different areas of rRPS invasion, which are of clinical significance and have application value.

Qi et al. (31) confirmed that the SARPS was directly connected to the bare area of the liver by dual-source CT. Because the SARPS is close to the diaphragm, radiofrequency ablation for HCC invading the diaphragm is not effective in achieving technical success and local tumor control (32). Therefore, TACE treatment of HCC invading this region is of particular clinical importance. Our study demonstrated that the most frequent collateral blood supply vessel was the RIPA, followed by the RAA and RRCA, which were the second and third most frequent EHCs, respectively. The tumors were supplied by the HA alone (*n* = 8) and both the HA and EHCs (*n* = 14) at the initial TACE procedure. As the number of TACE sessions increased, the number of tumors supplied by the HA alone gradually decreased, and the frequency of tumors supplied by EHCs increased. Blood supply from EHCs alone appeared in the first recurrence. EHCs such as the OA, RPICA, and SA rarely appeared in the later TACE sessions. The reason may be that repeated TACE exaggerates the development of such rare EHCs. These results are consistent with those of previous studies (16, 33), and the higher frequency of RAA appearance may be because the right superior adrenal artery originates from the RIPA, which is often overlooked.

The RAPS contains many important organs, such as the duodenum, pancreas, retroperitoneal segments of the ascending colon, the roots of the small bowel mesentery and transverse mesocolon (34), therefore, HCC invasion into the RAPS can adhere to other organs at an early stage and increase the degree of extrahepatic collaterals and early extrahepatic metastasis. We are less aggressive in these patients for fear of serious complications, despite the use of superselective embolization technology, which may affect the efficacy of TACE and patient survival. The incidence of tumor EHCs in the RAPS is similar to that in the SARPS, and it has also been confirmed that the RAPS is connected to the bare area of the liver by the right prerenal fascia (13, 31). However, we found that the OA appeared in the initial TACE procedure, and the appearance of the RCA and rare EHCs was higher in the RAPS than in the SARPS. This is consistent with the anatomy; moreover, this finding also indicated that HCC with RAPS invasion is more complex and difficult to treat with complete embolization and needs multiple TACE sessions or combined targeted therapy or other therapies.

Some limitations in our present study should be mentioned. First, this was a single-center, non-randomized and retrospective study with selection bias. Second, we did not use C-arm CT or guidance software in this study. C-arm CT and guidance software can improve the recognition of EHCs supplying the tumors (35), and we probably missed unsuspected EHCs in some patients. Thus, the prevalence of EHCs in this study may be underestimated. The third limitation is that the number of patients included in our study population was relatively small; thus, more independent predictors could have been identified in the Cox analysis if enough variables were included, and the study population needs to be enlarged in a follow-up study to confirm our findings.

In conclusion, the efficacy of TACE differs among HCC with different areas of rRPS invasion, and the tumors with rRPS invasion can be supplied by several EHCs. The RIPA, RRCA, and RAA are the common feeding vessels in the early stage, while other rare EHCs supply can be observed during the late stages. To improve the efficacy of TACE therapy, it is important for interventional radiologists to confirm the origin of EHCs for HCC with different areas of rRPS invasion.

## Data Availability Statement

The datasets generated for this study are available on request to DG, guodaj@hospital.cqmu.edu.cn.

## Ethics Statement

The studies involving human participants were reviewed and approved by Ethics Committee of The Second Affiliated Hospital of Chongqing Medical University. The patients/participants provided their written informed consent to participate in this study. Written informed consent was obtained from the individual(s) for the publication of any potentially identifiable images or data included in this article.

## Author Contributions

XLi, XLu, and DG designed the study. XLi and XLu performed the TACE procedures. XLi and DG evaluated the radiological images. XLi, GL, HC, and SC evaluated the clinical data. XLi, WS, and HZ analyzed the data. XLi and GL wrote the manuscript with input from all authors. All authors contributed to the article and approved the submitted version.

## Conflict of Interest

The authors declare that the research was conducted in the absence of any commercial or financial relationships that could be construed as a potential conflict of interest.
